# Arthroscopic Treatment for Femoroacetabular Impingement Syndrome with External Snapping Hip: A Comparison Study of Matched Case Series

**DOI:** 10.1111/os.13109

**Published:** 2021-06-17

**Authors:** Shan‐xing Zhang, Ming‐yang An, Zhong‐li Li, Zhi‐gang Wang, Yu‐jie Liu, Wei Qi, Chun‐bao Li

**Affiliations:** ^1^ Department of Orthopaedic Surgery The First Affiliated Hospital of Zhejiang Chinese Medical University Hangzhou China; ^2^ Department of Orthopaedics, The Fourth Medical Center Chinese PLA General Hospital Beijing China; ^3^ Medical School of Chinese PLA Beijing China

**Keywords:** External snapping hip, Femoroacetabular impingement syndrome, Hip arthroscopy, Iliotibial band (ITB) release, Outcome

## Abstract

**Objective:**

To determine the effectiveness of hip arthroscopy combined with endoscopic iliotibial band (ITB) release in patients with both femoroacetabular impingement (FAI) syndrome and external snapping hip (ESH).

**Methods:**

Retrospectively review the preoperative and minimum of 2‐year follow‐up data of patients with both FAI syndrome and ESH who underwent endoscopic ITB release during hip arthroscopy (FAI + ESH group) from January 2014 to December 2018. The same number of age‐ and gender‐matched FAI syndrome patients without ESH undergoing hip arthroscopy were enrolled in the control group (FAI group). Patient‐reported outcomes (PROs) including international Hip Outcome Tool (iHOT‐33), modified Harris Hip Score (mHHS), visual analog scale for pain (VAS‐pain), and abductive force of affected hip at 3 month and 2 years postoperatively were comparatively analyzed. The VAS‐satisfaction score of two groups at 2 years postoperatively were also analyzed.

**Results:**

The prevalence of ESH in FAI syndrome patients undergoing hip arthroscopy in our institution was 5.5% (39 of 715 hips), including nine males (10 hips) and 29 females (29 hips). The mean age at the time of surgery was 32.1 ± 6.9 years (range, 22–48 years). According to inclusion and exclusion criteria, 23 patients were enrolled in FAI + ITB group. Twenty‐three age‐ and sex‐matched FAI syndrome patients were enrolled in FAI group. At 24 months postoperatively, no patient still suffered ESH symptoms and painful palpation at lateral region in FAI + ITB group. The iHOT‐33, mHHS, and VAS‐pain score of patients in FAI + ESH group were significantly severer than patients in FAI group preoperatively (41.6 ± 7.5 *vs* 48.8 ± 7.2, 54.8 ± 7.2 *vs* 59.2 ± 6.9, 5.5 ± 0.9 *vs* 4.7 ± 1.0; *P* < 0.05), while there was no significant difference in these scores between the patients in FAI + ESH group and FAI group at 3‐month and 24‐month follow‐up (73.6 ± 8.5 *vs* 76.1 ± 6.9, 85.3 ± 7.8 *vs* 84.2 ± 6.6, 0.8 ± 0.9 *vs* 0.6 ± 0.9; *P* > 0.05). At 3 months after surgery, the abductive force of operated hip was significantly smaller than that in FAI group (82.4 ± 12.4 N *vs* 91.9 ± 16.1 N, *P* < 0.05), whereas there was no significant difference at 24 months after surgery (101.6 ± 14.9 N *vs* 106.5 ± 13.7 N, *P* > 0.05). The VAS‐satisfaction scores of patients in the two groups were at a similarly high level (90.5 ± 6.8 *vs* 88.8 ± 7.3, *P* > 0.05). There was no complication and no arthroscopic revision in either group until 2‐year follow‐up.

**Conclusion:**

Although abductive force recovery of the hip was delayed, hip arthroscopy combined with endoscopic ITB release addressed hip snapping in patients with both FAI syndrome and ESH, and could get similar functional improvement, pain relief, recovery speed, as well as patient satisfaction compared with the pure hip arthroscopy in FAI syndrome patients without ESH.

## Graphical abstract

This article confirmed that hip arthroscopy combined with endoscopic iliotibial band (ITB) release would address hip snapping in patients with both femoroacetabular impingement (FAI) syndrome and external snapping hip (ESH), and would get similar functional improvement, pain relief, recovery speed, as well as patient satisfaction, compared with the pure hip arthroscopy in FAI syndrome patients without ESH.

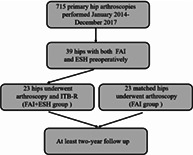

## Introduction

Femoroacetabular impingement (FAI) syndrome is defined as a dynamic impingement between the anterior femoral head–neck junction and anterior acetabular rim, causing chondral and labral damages as a consequence of repetitive hip motion^1^. The primary symptom of FAI syndrome is motion‐related or position‐related pain in the hip or groin. Pain may also be felt in the back, buttock, or thigh. In addition to pain, patients may also describe clicking, catching, locking, stiffness, restricted range of motion, or giving way^2^. FAI syndrome has been the most common cause of hip pain in active young and middle‐age adults, while it is also the most common indication for hip arthroscopy^1^, ^3^. According to the type of morphologic abnormality, FAI syndrome is categorized into cam (caused by an abnormally shaped femoral head, specifically one which has lost its sphericity^4^), pincer (excessive coverage of the acetabulum over a normally shaped femoral head^5^), and mixed, which has both cam and pincer deformities.

FAI syndrome can be treated by conservative care, rehabilitation, or surgery. Without treatment, symptoms of FAI syndrome will probably worsen over time. Currently, the keys of non‐operative treatment are to avoid the aggravating activity for a time, work on maintaining muscle strength, and judiciously use anti‐inflammatory drugs or intra‐articular injections of hyaluronic acid and steroid.

Surgical management of FAI is necessary for patients who have no response to non‐operative treatments. Open surgeries such has surgical dislocation of hip or anterior mini‐open surgery and hip arthroscopy have different advantages and disadvantages. The mini‐anterior approach increases visualization of the femoral head–neck junction and allows for shorter recovery times without the morbidity of a surgical dislocation and trochanteric osteotomy. These advantages come at the cost of limited access to posterior pathology and decreased global visualization of the socket and femoral head^6^.

Hip arthroscopy has been increasingly used as a minimally invasive surgical intervention to address symptomatic FAI syndrome for the past two decades. Labral repair or debridement, acetabulum trimming, and femoral head–neck osteoplasty are the major procedures to address the labral tear and prevent from abnormal contacting of the acetabular rim against the femoral neck during hip arthroscopic surgery. Many studies have established that hip arthroscopy can afford FAI syndrome patients excellent pain relief, functional improvement, and even returning to full activity^7^.

Our clinical practice showed that prevalence of external snapping hip (ESH) among FAI syndrome patients is not uncommon. Due to the complexity of the structures around the hip, some FAI syndrome patients have extra‐articular disorders, such as trochanteric bursitis, ESH, or internal snapping hip, along with intra‐articular injuries, such as labral tear and cartilage injury^8^. ESH is typically caused by an enlarged or tight posterior portion of the iliotibial band (ITB) and tight anterior border of the tendinous insertion of the gluteus maximus muscle, and is often associated with repetitive activities, trauma, mechanical hip alterations such as decreased angulation of femoral neck (coxa vara), slight rotation of the greater trochanter, hyperplasia of the trochanteric bursa, use of the ITB for reconstructive procedures of knees, tightness of the ITB, leg lengthening after total hip replacement, muscle fibrosis after intramuscular injection, and iatrogenic process after surgical procedures (fibrotic scar tissue after total hip replacement, excessive prominence of the greater trochanter and placement of the femoral component too laterally with angulation of the stem in relation to the long axis of the femur)^9^. During exercise or simply ordinary daily activities, the thickened ITB slides over and catches of the superior border of the greater trochanter of the femur as the flexion and extension of the hip, causing snapping around the greater trochanter^10^, ^11^. Continuous presentation of ESH always results in hip pain or local painful palpation that is due to inflammatory thickening of ITB and great trochanteric bursitis. Most cases of ESH can be treated conservatively, which includes stretching, hip abductor strengthening, activity modification, and anti‐inflammatory injection underneath the iliotibial band to quickly relieve the great trochanteric bursitis. For the refractory patients who do not respond well to the nonsurgical treatments, surgical intervention including open or endoscopic ITB release is a considerable alternative^9^, ^12^, ^13^, ^14^.

Currently, arthroscopic surgeons tend to perform endoscopic ITB release when intra‐articular pathologies are addressed for the patients with both FAI syndrome and trochanteric bursitis and acceptable outcomes were reported^15^, ^16^, but a consensus is still lacking on treating for patients with both for FAI syndrome and ESH.

Therefore, the purpose of this study was to investigate: (i) whether ITB release during hip arthroscopy can address the hip snapping in patients with both FAI syndrome and ESH; (ii) whether patients with both FAI syndrome and ESH undergoing hip arthroscopy combined with endoscopic ITB release during hip arthroscopy would gain the same excellent outcome as those FAI syndrome patients without ESH undergoing pure hip arthroscopy surgery; (iii) whether ITB release during hip arthroscopy would affect abductive force of suffered hip.

## Methods

### Patient Selection

This study was a retrospective review of patients that underwent hip arthroscopy in our institution by one senior surgeon from January 2014 to December 2018. The research was approved by Institutional Review Board of our institution.

Inclusion criteria for the study were: (i) patients who had medical history, physical examination and radiographic findings consistent with FAIS. In addition, the patients also had ESH symptoms including snap by palpation of the trochanteric region during flexion and/or extension of the affected hip (with or without painful palpation of the trochanteric region); (ii) failed conservative treatment such as oral medication, intra‐articular injection, or physiotherapy for more than 3 months, and underwent hip arthroscopy combined with endoscopic ITB release at primary surgery; (iii) the same number of age‐ and gender‐matched FAI syndrome patients without ESH undergoing pure hip arthroscopy during same period were enrolled in control group; (iv) the main evaluation indicators included symptom of hip snapping, international Hip Outcome Tool (iHOT‐33), modified Harris Hip Score (mHHS), Visual Analog Scale (VAS)‐pain and VAS‐satisfaction; (v) this study was a retrospective case–control study.

Exclusion criteria were: (i) the affected hip had suffered acute severe trauma before surgery and patients underwent revision or bilateral hip arthroscopy; (ii) followed‐up for less than 2 years; (iii) patients had history of congenital or pediatric deformities (developmental dysplasia of the hip [DDH], slipped capital femoral epiphysis, Legg‐Calve‐Perthes disease, etc.); (iv) osteoarthritis of the affected hip was severer than Tönnis grade 2; (v) patients that had lumbosacral disease, deep gluteal syndrome, or tear/calcification of gluteus medius.

When the patients with both FAI syndrome and ESH were going to undergo surgical treatment, they were offered two surgery plans for selection: hip arthroscopy combined with endoscopic ITB release; hip arthroscopy without ITB release, and the ESH would be left for further conservative treatments. All the detailed information about these two surgical treatments would be introduced carefully and the final operation—hip arthroscopy with or without ITB release—was determined by patients themselves.

Patients with both FAI syndrome and ESH undergoing hip arthroscopy combined with endoscopic ITB release meeting the inclusion criteria but not meeting the exclusion criteria were enrolled in FAI + ESH group. For comparison, the same number of age‐ and gender‐matched FAI syndrome patients without ESH undergoing pure hip arthroscopy during the same period and also not meeting the exclusion criteria were enrolled in FAI group as control group.

### Imaging Testing and Measurements

Plain radiographs of all patients including anteroposterior pelvic view and Dunn view were reviewed^17^. The lateral center edge (LCE) angle of Wiberg and joint space width at its lowest point was measured from the anteroposterior pelvic view; alpha angle and off‐set in millimeters (mm) was measured from the Dunn view, by three independent orthopaedic surgeons with a computer picture archiving and measurement system^1^. Patients' classification of FAI syndrome was defined based on the results of imaging measurements^1^. Cam impingement was defined as an alpha angle greater than 50° and/or off‐set less than 7.2 mm. Pincer impingement was defined as that presenting of crossover sign, coxa profunda, or protrusioacetabuli^2^.

Three‐dimensional computed tomography (3D‐CT) scanning was performed routinely to specifically localize the pincer or cam deformity. Magnetic resonance imaging (MRI) was obtained for all patients to evaluate the presence of labral and chondral injuries and bursitis around the greater trochanter of femur^18^.

### Surgical Technique

#### Hip Arthroscopy

The intra‐articular operation was performed before the ITB release during surgery in patients with both FAI syndrome and ESH.

#### Anesthesia and Position

After successful general anesthesia, patients were positioned supine on a traction table, with both feet in well‐padded traction boots and the patient's groin in full contact with the perineal post. It is necessary to check the genitalia, particularly in male patients, to avoid direct compression during the traction process. Once the surgical area was draped, traction was applied using the fine traction adjustment of the traction table until the hip is distracted approximately 8 to 10 mm, checking by fluoroscopy.

#### Approach and Operation in Central Compartment

**Fig. 1:**
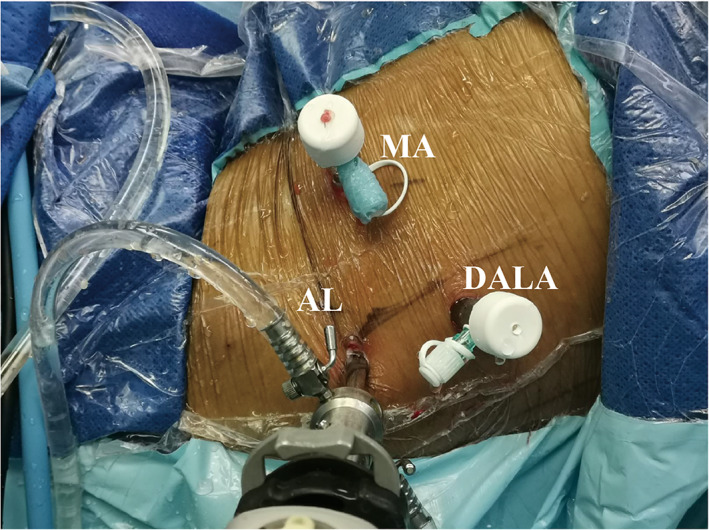
Intra‐operative photograph of a right hip. A standard anterolateral (AL), mid‐anterior (MA) and distal anterolateral portal approach (DALA) portals have been established for hip arthroscopy and endoscopic iliotibial band (ITB) release.

At this point, standard anterolateral (AL) and modified mid‐anterior (MA) portals were established. Using a beaver blade (Smith & Nephew, London, UK) or radiofrequency ablator (Smith & Nephew, London, UK), inter‐portal capsulotomy was created with attention to remain in the plane between the labrum and femoral head. Diagnostic arthroscopy was performed and once the labral tear was confirmed, the teared labral connecting edge of the acetabulum (mostly located at the anterolateral edge of the acetabulum) was exposed by debriding the portion of attachment of capsule on acerabulum rim with a radio frequency blade. Acetabuloplasty was performed with a 4.5 mm bur (Smith & Nephew, London, UK) to correct pincer deformities. Then the distal anterolateral approach (DALA) was established 5 to 7 cm distal to AL portal and was used as access for anchors (Smith & Nephew, London, UK) for labral fixation^19^ (Fig. 1). When the labral tear was too severe or calcified, selective debridement until a stable labrum remained.

#### Operation in Peripheral Compartment

After the central compartment pathology has been addressed, the traction was released and the camera was positioned in the modified MA portal for viewing distally along the head–neck junction. Radio‐frequency blade through DALA portal was use to perform T‐capsulotomy at the anterior head–neck junction to expose the cam deformity. Then femoral osteochondroplasty was performed, using 5.5 mm bur, to remove the cam and restore the native head–neck offset. The position of the hip was changed by flexing, extending, and internal/external extending rotation of the hips to facilitate the exposure of the cam and the osteochondroplasty. Fluoroscopic guidance and arthroscopic examination under imaging were used to confirm the absence of residual bony impingement. Closure of the capsule was performed routinely in all patients.

### Endoscopic ITB Release

**Fig. 2:**
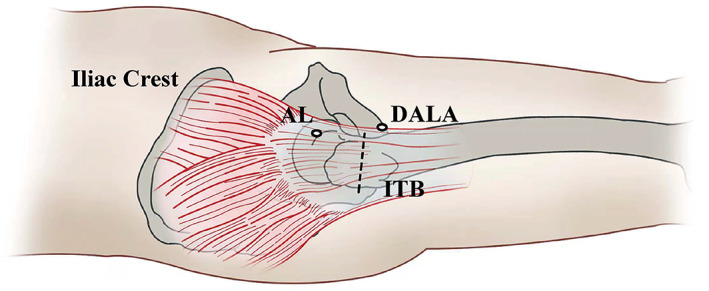
Transverse release (dashed line) of the iliotibial band (ITB) through anterolateral (AL) and distal anterolateral portal approach (DALA) portals (right hip).

**Fig. 3:**
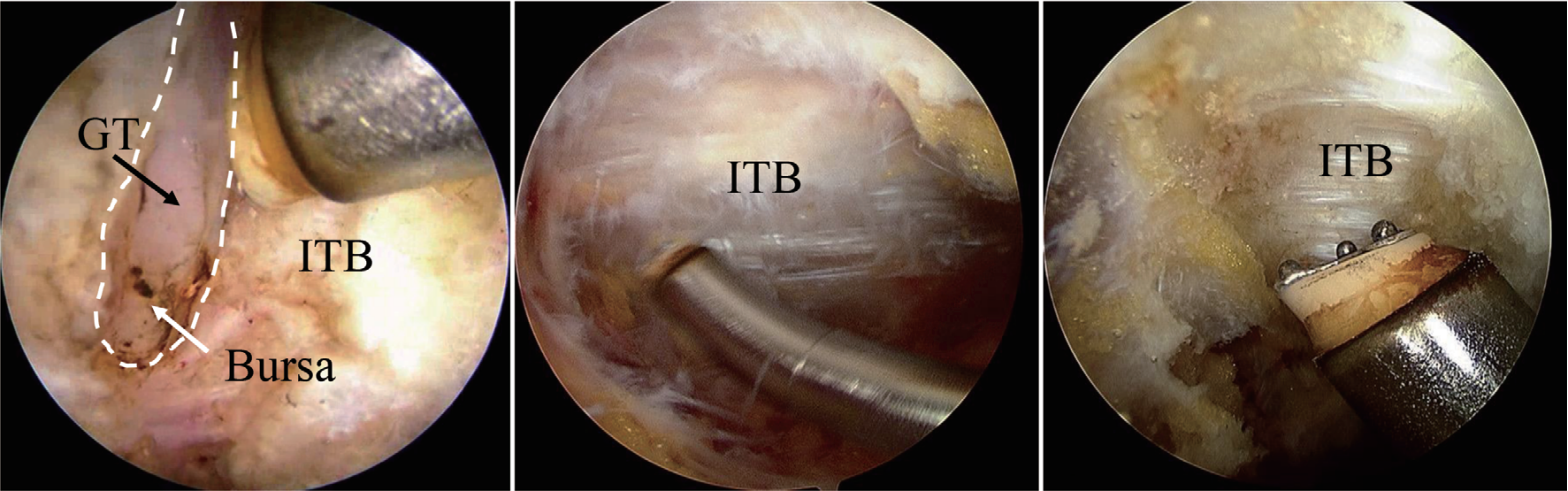
(A) Right hip of case 1: Iliotibial band (ITB) is transversal incised with radiofrequency blade. Bursa and greater trochanter (GT) are exposed. (B) Right hip of Case 2: ITB is exposed before releasing (C) Case 3. Thickened ITB is exposed and incised.

In the patients with both FAI syndrome and ESH, endoscopic ITB release was performed after the intra‐articular pathologies were addressed. With the hip in extension, the AL portal was used as viewing portal, and the DALA portal was used as operating portal to bluntly dissect and clean the overlying soft tissues on the ITB with a shaver. Hemostasis was very critical for maintaining a clear view as the inflammatory tissue was likely bleeding. Once the anterior and posterior border of the IBT was confirmed, transversal incision was performed with radiofrequency blade at the prominent of the greater trochanter^20^. The transversal incision of ITB allowed for direct visualization of the bursa around greater trochanter as the proximal and distal flaps of incised ITB would translate proximally and distally, respectively, from the pull of residual tension afford by ITB. For the patients with severe bursitis, the inflammatory tissue around the greater trochanter would be debrided (Figs 2 and 3).

### Postoperative Rehabilitation

Patients were prescribed 200 mg of celecoxib twice daily for pain relief and prevention of heterotopic ossification in the first 4 weeks after surgery.

Postoperative rehabilitation in our center included the following. Physical therapy began postoperative day 1 with a protocol specific to the procedure performed. The day after surgery, patients were instructed on partial weight‐bearing walking using two crutches as long as it was tolerable. Patients also began ankle pump exercises and isometric contraction exercises of the gluteus medius muscle, waist and back muscles, and quadriceps. The hip joint was passively flexed up to 90°, with restriction on the eversion, external rotation, and backward extension.

In the patients who also underwent endoscopic ITB release, the hip joint was passively adducted to no more than 20° with the hip in extension. In both FAI syndrome patients and the patients with FAI syndrome and ESH, hip adduction and flexion were limited to a 20‐pound weight‐bearing restriction on the operated extremity for 2–4 weeks, depending on the management on the labrum. Patients were allowed to do full weight‐bearing walking 4 weeks after surgery, and the normal functional activities of the lower limb was restored. Patients could begin exercises such as jogging and stair climbing.

### Outcome Evaluation

#### iHOT‐33

Patient‐reported outcomes (PROs), including iHOT‐33 and mHHS, were employed for assessment of hip function. The iHOT‐33 consists of 33 items covering four domains: symptoms and functional limitations, sports and recreational activities, job‐related concerns, and social, emotional, and lifestyle concerns^21^. Each question is scored out of 100, with 0 representing the worst possible quality‐of‐life score and 100 representing the best. Totaling the scores from all questions answered and then dividing by the number of questions determined the patient's final score out of 100.

#### mHHS

The mHHS was designed by Byrd *et al*.^22^ in 2000 and contains eight questions covering three domains: pain, function, and activities of daily living. Like the original form, this version gives a score from 0 (extreme symptoms) to 100 (no symptoms).

#### VAS‐Pain and VAS‐Satisfaction

VAS methodology was used to assess patient‐determined hip pain and satisfaction. Using a ruler, the score of VAS‐pain was determined by measuring the distance (cm) on the 10‐cm line orientated from the left (0 mm indicating no pain or dissatisfaction at all) to the right (100 mm indicting extreme amount of pain or satisfaction) between the “0” anchor and the patient's mark, providing a range of scores from 0–10 for VAS‐pain and 0–100 for VAS‐satisfaction. A higher score indicates greater pain intensity or higher satisfaction with treatment.

#### Abductive Force of Hip

The abductive force of the affected hip was tested by a tension tester (WEIDU. Wenzhou, Zhejiang, China) fixed at ankle in lateral decubital position.

The iHOT‐33 score, mHHS, VAS‐pain score, and the abductive force in the two groups at preoperative, 3‐months and 2‐year postoperative follow‐up were collected. VAS‐satisfaction score of patients at 2‐year postoperative follow‐up was also collected.

### Statistical Analysis

Calculations were carried out with SPSS (version 22.0, SPSS, Archimonde, New York, USA) software package. All data were presented as mean ± SD. Logistic regression was used for testing for normal distribution. The unpaired Student's *t*‐test for independent samples was used for comparison of age, BMI, symptom duration, radiographic measurements, abductive force, and patient‐reported outcome scores such as iHOT‐33, mHHS, VAS‐pain, and VAS satisfaction of two groups. The difference of constitution of gender and FAI type in the two groups was analyzed with chi‐square test (Pearson test). All reported *P* values are two‐tailed, with an alpha level of 0.05 indicating significance.

## Results

### General Results

**Fig. 4:**
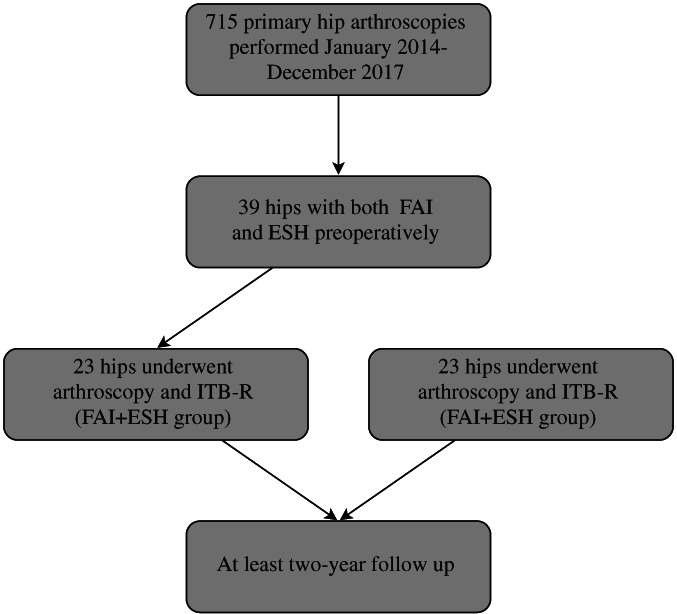
Flow chart illustrating the full patient selection process.

The data of 623 FAI syndrome patients (715 hips) who underwent hip arthroscopy during January 2014 to December 2018 by one surgeon were reviewed. Of these patients, there were 38 patients (39 hips) who also suffered ESH preoperatively, including nine males (10 hips) and 29 females (29 hips). The prevalence of ESH in this special cohort of FAI hips was 5.5% (39 hips of 715 hips). There were 23 patients who underwent endoscopic ITB release during hip arthroscopy and also met the inclusion criteria but did not meet the exclusion criteria (FAI + ESH group, Fig. 4).

Twenty‐three age‐ and gender‐matched FAI syndrome patients without ESH who underwent hip arthroscopy and also did not meet the exclusion criteria were enrolled in FAI group.

The age, gender, BMI, FAI types, body mass index (BMI), symptom duration of FAI syndrome, and the radiographic measurements are listed in Table 1. There was no significant difference of these items between the patients in the two groups.

**TABLE 1:** Patient demographics and preoperative radiographic measurements for the FAI + ESH group and FAI group (mean ± SD)

Demographics	FAI + ESH group (*n* = 23)	FAI group (*n* = 23)	*t/F* value	*P* value
Age (years)	32.9 ± 8.0	33.3 ± 7.6	*t* = 0.17	0.865
Gender (male/female)	4 /19	4 /19	—	—
Body mass index (kg/m^2^)	22.1 ± 2.9	21.6 ± 2.1	*t* = 0.551	0.584
Symptom duration of FAI (m)	14.5 ± 7.8	18.5 ± 8.3	*t* = 1.684	0.099
Symptom duration of ESHS (m)	23.5 ± 17.9	—	—	—
FAI type (cases)				
Cam	15	14		
Pincer	4	5	*F* = 0.256	1
Mixed	4	4		
Radiographic measurements				
Lowest joint space (mm)	4 ± 0.5	3.9 ± 0.6	*t* = 0.748	0.458
Alpha angle (°)	59.7 ± 5.7	60.9 ± 6.6	*t* = 0.691	0.493
Off‐set (mm)	5.3 ± 1.2	5.5 ± 1.3	*t* = 0.694	0.491
LCE angle (°)	32.3 ± 7.0	34.7 ± 6.8	*t* = 1.201	0.236

BMI, Body mass index; ESH, external snapping hip; FAI, femoroacetabular impingement; LCE angle, lateral center edge angle.

### Outcomes

#### Snapping of Hip

At 3 months postoperative and 24 months postoperative, no patient still suffered ESH symptom and painful palpation at lateral region of operated hip in FAI + ESH group.

#### iHOT‐33, mHHS, and VAS‐Pain

The mean value of iHOT‐33, mHHS, VAS‐pain of patients in two groups at preoperative, 3‐month follow‐up, and 24‐month follow‐up were listed in Table 2. The iHOT‐33, mHHS, and VAS‐pain score of patients in FAI + ESH group were significantly better than patients in FAI group preoperatively (41.6 ± 7.5 *vs* 48.8 ± 7.2, 54.8 ± 7.2 *vs* 59.2 ± 6.9, 5.5 ± 0.9 *vs* 4.7 ± 1.0; *P* < 0.05), while there was no significant difference of these scores between the patients in FAI + ESH group and FAI group at 3‐month follow‐up and 24‐month follow‐up (3‐month 54.6 ± 7.7 *vs* 57.8 ± 6.6, 67.7 ± 6.9 *vs* 68.9 ± 5.5, 2.3 ± 0.7 *vs* 2 ± 0.8; *P* > 0.05; 24‐month: 73.6 ± 8.5 *vs* 76.1 ± 6.9, 85.3 ± 7.8 *vs* 84.2 ± 6.6, 0.8 ± 0.9 *vs* 0.6 ± 0.9; *P* > 0.05).

**TABLE 2:** Outcomes of the patients in FAI + ESH group and FAI group (mean ± SD)

Outcomes	FAI + ESH group	FAI group	*t* value	*P* value
iHOT‐33: preoperation	41.6 ± 7.5	48.8 ± 7.2	3.364	0.002
iHOT‐33: postop‐3 m	54.6 ± 7.7	57.8 ± 6.6	1.526	0.134
iHOT‐33: postop‐24 m	73.6 ± 8.5	76.1 ± 6.9	1.105	0.275
mHHS: preoperation	54.8 ± 7.2	59.2 ± 6.9	2.107	0.041
mHHS: postop‐3 m	67.7 ± 6.9	68.9 ± 5.5	0.638	0.527
mHHS: postop‐24 m	85.3 ± 7.8	84.2 ± 6.6	0.509	0.613
VAS‐pain: preoperation	5.5 ± 0.9	4.7 ± 1	2.56	0.014
VAS‐pain: postop‐3 m	2.3 ± 0.7	2 ± 0.8	1.558	0.126
VAS‐pain: postop‐24 m	0.8 ± 0.9	0.6 ± 0.9	0.658	0.514
AF (N): postop‐3 m	82.4 ± 12.4	91.9 ± 16.1	2.228	0.031
AF (N): postop‐24 m	101.6 ± 14.9	106.5 ± 13.7	1.162	0.252
VAS‐satisfaction	90.5 ± 6.8	88.8 ± 7.3	0.811	0.422

AF, Abductive force; iHOT‐33, international Hip Outcome Tool‐33; m, months; mHHS, modified Harris Hip Score; VAS, visual analog scale.

#### Abductive Force of Hip

At 3 months after surgery, the abductive force of operated hip in FAI + ESH group was smaller than that in FAI group (82.4 ± 12.4 N *vs* 91.9 ± 16.1 N, *P* < 0.05), whereas there was no significant difference at 24 months after surgery (101.6 ± 14.9 N *vs* 106.5 ± 13.7 N, *P* > 0.05; Table 2).

#### VAS‐Satisfaction

The VAS‐satisfaction score of patients who underwent hip arthroscopy combined with ITB release during primary surgery was similarly high compared to those FAI patients who only underwent hip arthroscopy (90.5 ± 6.8 *vs* 88.8 ± 7.3, *P* > 0.05; Table 2).

### Complication and Revision

There was no complication and no arthroscopic revision in both groups until 2‐year follow‐up.

## Discussion

### Summary of This Study

By reviewing the data of FAI syndrome patients undergoing hip arthroscopy in our institution, we found that the prevalence of ESH in FAI syndrome patients in this special cohort was 5.5% (39 in 715 hips). Our data also indicate that females were more likely to suffer FAI syndrome and ESH concurrently, and patients with both FAI syndrome and ESH showed severer pain and dysfunction than those who just suffered FAI syndrome. We also found that patients with both FAI syndrome and ESH undergoing hip arthroscopy combined with ITB release would address the hip snapping and achieve excellent outcomes, presenting as similarly considerable level of function improvement, pain relief, and patient satisfaction, without compromise of abductive strength of the hip joint.

### Pathogenesis of ESH in FAI Syndrome Patients

Due to the complex structure around the hip, extra‐articular disorders such as hip snapping and trochanter bursitis are still catching the interesting of arthroscopic surgeon as these conditions may affect the outcome of the hip arthroscopy in FAI syndrome patients. Besides the enlarged or tight posterior portion of the iliotibial band (ITB) and tight anterior border of the tendinous insertion of the gluteus maximus muscle, mechanical hip alterations that change the normal relationship between ITB and the great trochanter also play the critical factor in resulting in ESH, such as decreased angulation of femoral neck, rotation of the greater trochanter, as well as tightness of the ITB^9^.

Greater trochanteric disorders such as ESH and trochanteric bursitis have a high prevalence in high‐activity patients^23^. Pozzi *et al*.^24^ observed an overall 16.4% rate of trochanteric bursitis in patients who underwent magnetic resonance arthrography (MRA) of the hip for a suspected FAI. FAI syndrome patients are more likely to get greater trochanteric disorders due to their underlying hip condition, altered gait mechanics, pelvic muscular imbalance, and lumbopelvic abnormality^16^, ^24^, ^25^. The incidence rate of EHS in the FAI cohort which underwent hip arthroscopy in our institution was 5.5%, a little lower than Vap's research, which reviewed a cohort of 1278 patients who underwent hip arthroscopy in their institution and showed a 7% prevalence of trochanteric bursitis in FAI patients^16^. In addition, our study showed that females comprised 76.3% of these patients with both FAI syndrome and ESH, which was consistent with the research reported previously.

ESH would cause lateral hip pain and psychological discomfort during daily activity when the hip is fully extended from in flexion and that would aggravate the underlying hip condition of patients who also had FAI syndrome, such as altered gait mechanics and pelvic muscular imbalance^13^, ^26^. As such, patients with FAI syndrome and ESH showed worse joint function and higher pain scoring. However, the patients with FAI and trochanteric bursitis had similar functional rating in Vap's research^16^.

### Therapeutic Strategy for FAI Syndrome with ESH Using ITB Release Technique

There is no consensus about the optimal therapeutic strategy for patients suffering ESH and FAI syndrome. ESH usually needs to be treated step by step according to the patient's condition. Primary interventions for ESH consist of rest, avoidance of those activities which would induce the external snapping. Whereas the snapping causes lateral hip pain and negatively affects daily activities, local injection of steroid, oral anti‐inflammatory medication, and ITB stretching can be used to alleviate pain and improve hip function. For the refractory cases, surgical ITB release is final option to address the pathology. Previous study confirmed that endoscopic ITB release can relieve external hip pain and make snapping disappear with minor invasion and low recurrence rate of snapping^13^, ^20^, ^27^. Endoscopic ITB release seems to have become the most favorable operative treatment for ESH, as surgical ITB release not only relieves lateral hip pain at great trochanter, but also thoroughly addresses the snapping. As a factor in the recovery of hip arthroscopy for FAI syndrome, endoscopic ITB release should be performed during hip arthroscopic surgery to prevent hip snapping for the FAI syndrome patient who also suffers ESH. Some surgeons mentioned that they performed ITB release after intra‐articular operation in patients who also suffered greater trochanteric disorders such as ESH or trochantericbursitis^15^, ^16^. Vap *et al*.^16^ also confirmed that patients with FAI and trochanteric bursitis undergoing bursectomy and ITB release would achieve as good result as the hip arthroscopy in the FAI syndrome patients without ESH. In our study, the patients with both FAI syndrome and ESH who underwent hip arthroscopy combined with ITB release achieved similar results of iHOT‐33, mHHS, and VAS‐pain scoring to the FAI patients without ESH undergoing arthroscopy after 3 month and 2 years, showing that arthroscopy combined with ITB release for addressing FAI syndrome and ESH simultaneous during primary surgery could afford similar and considerable recovery speed and midterm outcome to pure hip arthroscopy for FAI syndrome patients without ESH.

The technique of ITB release is still developing and surgeons have preferred endoscopic to open surgery for ITB release in recent years. Mitchell *et al*.^28^ reported an endoscopic ITB release technique, by performing cruciate incision on ITB, 2 cm horizontally and 2 cm longitudinally, to release the tight ITB and expose the trochanteric bursa. However, we used to perform a comparatively larger transversal incision to make a large defect on ITB to release the tight ITB. Normally a 5 to 7 cm incision can make the ITB thoroughly released and perfectly expose the trochanteric bursa. This transversal incision technique was openly performed in the 1980s and achieved very good outcomes^29^, ^30^. Liu *et al*.^31^ confirmed that ITB transversal incision for gluteus contracture had no obvious influence on the abductive force of the affected low extremity. In 2013, Zini *et al*.^20^ first reported that transversal incision technique for ITB release was safe and reproducible in ESH. Up until now, there is no evidence of which technique is better^9^. The reason we use transversal incision technique is that this method can easily release ITB completely, not like the cruciate incision technique, where the cross‐over point of vertical and horizontal incision should be at an appropriate location. Additionally, transversal incision diminishes the concern of residual inflammation caused by waggling of ITB tissue blades generated by cruciate incision. It also should be emphasized that it is not necessary to establish another distal portal which is usually used in isolated endoscopic ITB release surgery, as the DALA portal is absolutely enough to perform the ITB release.

### The Abductive Strength of Operated Hip After ITB Release

One of the most critical concerns of ITB release is the possibility of compromise of abductive strength. As far as we know, the cruciate incision or transversal incision technique reported in previous studies had no obvious affection on abductive force of the hip^14^, ^20^, ^28^. In our study, the mean abductive force of affected hips in FAI + ESH group was smaller than that in FAI group 3 months after surgery, but was similar at 2 years after surgery, indicating that ITB release during primary hip arthroscopy would delay the recovery of abductive force of the affected hip but would not have a final negative effect on it.

### Limitations of This Study

There are several limitations to this study. Firstly, the study was performed retrospectively using prospectively collected and follow‐up data, and as such is limited by the inherent limitations of retrospective studies. In addition, the age‐ and gender‐matched control group may could not show the advantage of ITB release during hip arthroscopy compared with arthroscopy without ITB release but followed by conservative treatment for ESH. Furthermore, the cohort in our single‐center study was small and may be unrepresentative of the general population due to the specific background of our institution. These limitations may mean that this study has selection bias and/or cannot generate enough power to reach statistical significance. Multi‐center prospective control study is necessary in the future.

### Conclusion

Although abductive force recovery of the hip was delayed, hip arthroscopy combined with endoscopic ITB release would address hip snapping in patients with both FAI syndrome and ESH, and would get similar functional improvement, pain relief, recovery speed, and patient satisfaction compared with the pure hip arthroscopy in FAI syndrome patients without ESH.
